# Detecting Mutations in the *Mycobacterium tuberculosis* Pyrazinamidase Gene *pncA* to Improve Infection Control and Decrease Drug Resistance Rates in Human Immunodeficiency Virus Coinfection

**DOI:** 10.4269/ajtmh.15-0711

**Published:** 2016-12-07

**Authors:** Matthew Z. Dudley, Patricia Sheen, Robert H. Gilman, Eduardo Ticona, Jon S. Friedland, Daniela E. Kirwan, Luz Caviedes, Richard Rodriguez, Lilia Z. Cabrera, Jorge Coronel, Louis Grandjean, David A. J. Moore, Carlton A. Evans, Luz Huaroto, Víctor Chávez-Pérez, Mirko Zimic

**Affiliations:** 1 Department of International Health, Johns Hopkins Bloomberg School of Public Health, Baltimore, Maryland; 2 Laboratorio de Bioinformática y Biología Molecular, Laboratorios de Investigación y Desarrollo, Facultad de Ciencias y Filosofía, Universidad Peruana Cayetano Heredia, Lima, Peru; 3 Laboratorio de Investigación en Enfermedades Infecciosas, Laboratorios de Investigación y Desarrollo, Facultad de Ciencias y Filosofía, Universidad Peruana Cayetano Heredia, Lima, Peru; 4 Asociación Benéfica Proyectos en Informatica, Salud, Medicina, y Agricultura (PRISMA), Lima, Peru; 5 Hospital Nacional Dos de Mayo, Lima, Peru; 6 Facultad de Medicina, Universidad Nacional Mayor de San Marcos, Lima, Peru; 7 Infectious Diseases and Immunity, Wellcome Trust Centre for Global Health Research, Imperial College London, London, United Kingdom; 8 Department of Medical Microbiology, St. George's Hospital, London, United Kingdom; 9 Infections Diseases and Immunity, Imperial College London, London, United Kingdom; 10 Hospital de Apoyo Maria Auxiliadora, Lima, Peru; 11 TB Centre, London School of Hygiene and Tropical Medicine, London, United Kingdom; 12 Innovation For Health and Development (IFHAD), Laboratory of Research and Development, Universidad Peruana Cayetano Heredia, Lima, Peru; 13 Universidad Nacional Mayor de San Marcos, Lima, Peru

## Abstract

Hospital infection control measures are crucial to tuberculosis (TB) control strategies within settings caring for human immunodeficiency virus (HIV)–positive patients, as these patients are at heightened risk of developing TB. Pyrazinamide (PZA) is a potent drug that effectively sterilizes persistent *Mycobacterium tuberculosis* bacilli. However, PZA resistance associated with mutations in the nicotinamidase/pyrazinamidase coding gene, *pncA*, is increasing. A total of 794 patient isolates obtained from four sites in Lima, Peru, underwent spoligotyping and drug resistance testing. In one of these sites, the HIV unit of Hospital Dos de Mayo (HDM), an isolation ward for HIV/TB coinfected patients opened during the study as an infection control intervention: circulating genotypes and drug resistance pre- and postintervention were compared. All other sites cared for HIV-negative outpatients: genotypes and drug resistance rates from these sites were compared with those from HDM. HDM patients showed high concordance between multidrug resistance, PZA resistance according to the Wayne method, the two most common genotypes (spoligotype international type [SIT] 42 of the Latino American-Mediterranean (LAM)-9 clade and SIT 53 of the T1 clade), and the two most common *pncA* mutations (G145A and A403C). These associations were absent among community isolates. The infection control intervention was associated with 58–92% reductions in TB caused by SIT 42 or SIT 53 genotypes (odds ratio [OR] = 0.420, *P* = 0.003); multidrug-resistant TB (OR = 0.349, *P* < 0.001); and PZA-resistant TB (OR = 0.076, *P* < 0.001). In conclusion, *pncA* mutation typing, with resistance testing and spoligotyping, was useful in identifying a nosocomial TB outbreak and demonstrating its resolution after implementation of infection control measures.

## Introduction

Sound hospital infection control measures are critical for preventing and managing nosocomial tuberculosis (TB) outbreaks.^1^,^2^ This is especially true in wards caring for people living with human immunodeficiency virus (HIV) infection due to the heightened risk of TB among immunosuppressed patients.^3^ Compounding this problem is the growing threat of multidrug-resistant tuberculosis (MDRTB), defined as strains of TB resistant to rifampin and isoniazid, because patients living with HIV infection and MDRTB infection have extremely high mortality and require complicated and expensive treatment.^4^,^5^

Despite having a successful community-based national TB program since the 1990s,^6^ hospital TB infection control in Peru has proven challenging.^7^ Peruvian national surveillance data from 2012 estimated that 3.9% of new TB cases were caused by MDRTB; only 18% of TB patients knew their HIV status, and only 17% of patients living with HIV infection were tested for TB.^8^ In Lima, Peru's capital, a study by Campos and others found that 35 of 81 (43%) HIV/TB coinfected patients sampled from 10 local hospitals between 1999 and 2000 were infected with MDRTB.^9^

Pyrazinamide (PZA) resistance is another important factor in TB control. PZA facilitates eradication of persistent bacteria and thus its incorporation into drug regimens is key to shortening treatment duration.^10^–^12^ PZA resistance has been shown to be associated with mutations in the pyrazinamidase (PZAse) coding gene, *pncA*.^13^,^14^

In the HIV ward of Lima's Hospital Dos de Mayo (HDM), we studied the utility of *pncA* mutation typing, in tandem with phenotypic resistance testing and spoligotyping, to detect nosocomial outbreaks and inform the hospital's new infection control practices.

## Methods

### Study design.

#### Patient recruitment.

An anonymized TB strain bank was compiled from clinical research studies that enrolled adults diagnosed with pulmonary TB in Lima, Peru, between 1999 and 2005. A total of 794 study patient primary isolates (one per patient) with confirmed culture-positive TB infection were opportunistically spoligotyped from the following four cohorts: 1) 241 of 282 study inpatients from the HDM HIV unit, 2) 329 of 385 study outpatients from Hospital Maria Auxiliadora, 3) all 155 unselected National TB Control Program study patients from 10 government clinics in north Lima, and 4) all 69 National TB Control Program study patients selected for high risk of TB or MDRTB from five government clinics in east Lima. Study recruitment has been previously reported,^5^,^15^–^17^ and all study protocols and consent forms were approved by the institutional review boards of Universidad Peruana Cayetano Heredia, Asociación Benéfica Proyectos en Informatica, Salud, Medicina, y Agricultura, Regional Ministry of Health of Lima, HDM, and/or the Johns Hopkins Bloomberg School of Public Health, and all participants gave informed, written consent.

#### Infection control.

Before 2001, all HIV-positive inpatients at HDM were admitted to a ward constituting one large room measuring 35 × 8 × 4.5 m height. Infection control measures consisted primarily of large windows that facilitated natural ventilation, and health-care workers in direct contact with patients were required to use protective N95 respirators. With the help of donor funds, a new respiratory isolation ward for all HIV-infected patients with suspected or confirmed TB opened in 2001, slightly over 2 years into the study, as part of an effort to improve the hospital's TB control measures. This ward separated these patients from their immunosuppressed HIV-positive TB-negative counterparts for the first time. The new ward was equipped with a mechanically ventilated negative pressure air filtration system. Additional control measures implemented concurrently included relocation of the waiting area for outpatients to an outdoor space in the adjacent gardens, and mandatory sputum smear microscopy for all patients with respiratory symptoms at the time of hospitalization. Further details outlining the design, implementation, impact, and cost-effectiveness of this intervention are presented in the accompanying article in this issue. Second-line drugs were not universally available at HDM until 2004. A separate study evaluating the effects of ultraviolet germicidal light on TB transmission was conducted from 2002 to 2004 in the isolation ward. For the duration of this study, these lights were switched off and on for alternate 24-hour periods.^18^

It is important to note that although we discuss our data before and after this infection control intervention in both the HIV-positive HDM inpatients and the HIV-negative outpatients from other sites, the intervention only took place in the HIV-positive HDM inpatients, whereas there was no infection control intervention in the HIV-negative outpatients from other sites whose data are used solely for simultaneous comparison of prevalence.

#### Defining drug resistance.

Spoligotyping data were available for all isolates. Multidrug resistance was determined by microplate colorimetric^19^ and microscopic-observation drug-susceptibility (MODS)^15^,^16^,^20^ assays. Multidrug resistance data were unavailable for 12 isolates due to issues with the growth of the isolate in these assays.

PZA resistance was determined by the standard Wayne test,^21^ the results of which were available for all but 193 isolates. Although the Wayne test is an indirect biochemical test, it was shown to be a good predictor of PZA resistance demonstrated via the BACTEC 460TB and MGIT960 systems (Becton Dickinson Diagnostics, Sparks, MD) in the 127 isolates for which these test results were available (Pearson's correlation coefficient, *R* = 0.74). In particular, the Wayne test accurately predicted the PZA resistance of our two most prevalent mutations (13/14 in agreement for D49N and 4/4 in agreement for T135P) and our wild-type isolates (52/55 in agreement).

A total of 262 isolates, including all confirmed PZA-resistant strains and almost all MDR (131/135) strains, along with an equal number of randomly selected drug-susceptible strains, were tested for mutations in the *pncA* gene by DNA sequencing.^22^

### Data analysis.

Pearson's χ^2^ test for independence was used to compare categorical variables. Simple logistic regressions for both MDR and PZA resistance were performed for relevant independent variables such as genotype, HIV status, age, and sex. Best-fit models were created by backwards selection to include only those variables with statistical significance in both the simple and multiple models. Nested models were compared using the likelihood ratio test. Spoligotype was defined as an indicator variable to compare each of the five most prevalent genotypes—spoligotype international type (SIT) numbers 50, 53, 33, 42, and 1—with all other genotypes grouped together. The best-fit models were repeated with the addition of the interaction terms of HIV status on genotype to examine for potential effect modification. Odds ratios (ORs) were calculated using logistic regression and were adjusted for age and gender. All *P* values were two sided and *P* < 0.05 was considered statistically significant. Analysis was performed using STATA/IC 12.1 software (STATA Corp., College Station, TX).

## Results

### Patients.

A total of 794 study patients diagnosed with a new TB episode with a positive culture had their *Mycobacterium tuberculosis* strain spoligotyped.^17^ All 241 HDM patients were HIV seropositive (30% of total), and all other patients were HIV negative or of unknown HIV status (Table 1). The outpatients of unknown HIV status were assumed to be HIV negative for the purpose of this analysis because the prevalence of HIV infection in Peru was less than 0.5% of the general population, and 1.7% of TB patients.^5^

### Genotypes using SIT numbers.

Of these 794 isolates, 149 different spoligotypes were identified. The five most common TB genotypes among all patients were SIT 50 of the Harlem3 clade (16% of all isolates), SIT 53 of the T1 clade (12%), SIT 33 of the Latino American-Mediterranean (LAM)-3 clade (8.3%), SIT 42 of the LAM9 clade (7.4%), and SIT 1 of the Beijing clade (5.5%). Together, these five genotypes comprised 397 (50%) of the TB isolates (Table 1).

### Drug resistance.

PZA resistance, defined according to the Wayne test, was detected in 16% of isolates overall. Of the MDR isolates tested, 59% were also PZA resistant, compared with just 3.6% of non-MDR isolates tested (Table 1).

MDR and PZA resistance were strongly correlated (Pearson's correlation coefficient, *R* = 0.63). This correlation was seen even more clearly among HIV-positive inpatients (*R* = 0.79), compared with HIV-negative outpatients (*R* = 0.33).

Simple logistic regressions were performed to examine the associations of common genotypes, HIV status, age, and sex with drug resistance. Multiple logistic regression models were then created using the best-fit methods described in the data analysis section above. These revealed SIT 42 and SIT 53 to be the only genotypes to have statistically significant associations with both MDR and PZA resistance after adjusting for HIV status. In these models, SIT 42 had 12 times higher odds of being MDR and 12 times higher odds of PZA resistance than the less common genotypes, and SIT 53 had 3.8 times higher odds of being MDR and 5.0 times higher odds of PZA resistance. Patients living with HIV infection had 3.6 times higher odds of being MDR and 4.4 times higher odds of PZA resistance than patients without HIV infection, independent of the aforementioned genotypes (all *P* < 0.001) (Table 2).

Interaction terms between HIV and these genotypes were also explored but did not improve model fit. The model examining the interaction of SIT 42 and HIV coinfection on MDR was the only new model that retained some statistical significance (Table 2).

### *pncA* mutations.

Of the 262 TB isolates examined for *pncA* mutations, the two most common were G145A (a D49N PZAse mutation) and A403C (a T135P PZAse mutation), found in 12% and 5.3% of examined isolates, respectively. All 32 G145A isolates were of the SIT 42 genotype, and all were MDR and PZA resistant. Of these isolates, 31 were from the HDM HIV ward. Likewise, all 15 of the A403C isolates were of the SIT 53 genotype, MDR, and PZA resistant; 11 of these were from the HDM HIV ward (Table 3).

The only other common *pncA* mutation significantly associated with MDR and PZA resistance was C309G (a Y103Stop protein mutation), found in 3.4% of examined isolates. Eight of nine C309G isolates were MDR and PZA resistant, six of which were also SIT 53. All nine were from the HDM HIV ward (Table 3).

### ORs of genotype and drug-resistant infection after the implementation of the infection control intervention.

As described above, an isolation ward for HIV/TB coinfected patients was opened in the HIV unit of HDM 2 years into the study as an infection control intervention. Among HDM patients, the odds of disease caused by MDRTB after the intervention was reduced by almost two-thirds compared with the odds before the intervention (OR = 0.35, *P* < 0.001), and the odds of PZA-resistant TB disease after the intervention was reduced by over 90% compared with the odds before the intervention (OR = 0.076, *P* < 0.001). The odds of TB disease after the intervention caused by either SIT 42 or SIT 53, the genotypes with the strongest associations with drug resistance, was reduced by over half (OR = 0.420, *P* = 0.003). The odds of TB disease caused by SIT 50, the most common community strain, increased slightly after the intervention (OR = 1.729, *P* = 0.271), whereas the odds of disease caused by other common drug-susceptible community strains such as SIT 33 and SIT 1 were almost entirely unchanged (Table 4).

By comparison, among community patients, the odds of PZA-resistant TB disease after the intervention increased only slightly (OR = 1.3, *P* = 0.7), and the prevalence of each of the five most common genotypes (SIT 42, 53, 50, 33, and 1) remained relatively constant, changing by no more than 2.5% (Table 4). Of interest, however, is that the odds of MDRTB infection among community patients actually increased after the intervention by more than 3-fold (OR = 3.7, *P* = 0.04). This indicates that as MDRTB infection was becoming less common in the HIV ward, it was becoming increasingly common in the community.

### Prevalence of SIT 42 over time.

Before the intervention, the highly drug-resistant genotypes SIT 42 and SIT 53 were present in the HIV ward of HDM. In the first year of the study, prevalence of both SIT 42 and SIT 53 was much higher in HDM than in the community (15% versus 3.6% and 27% versus 12% of cases, *P* = 0.003 and *P* = 0.007, respectively). In the second year, the prevalence of SIT 42 in HDM increased dramatically to 39% of cases, whereas prevalence in the community stayed nearly the same at 3.8% of cases (*P* < 0.001).

**Figure 1.**
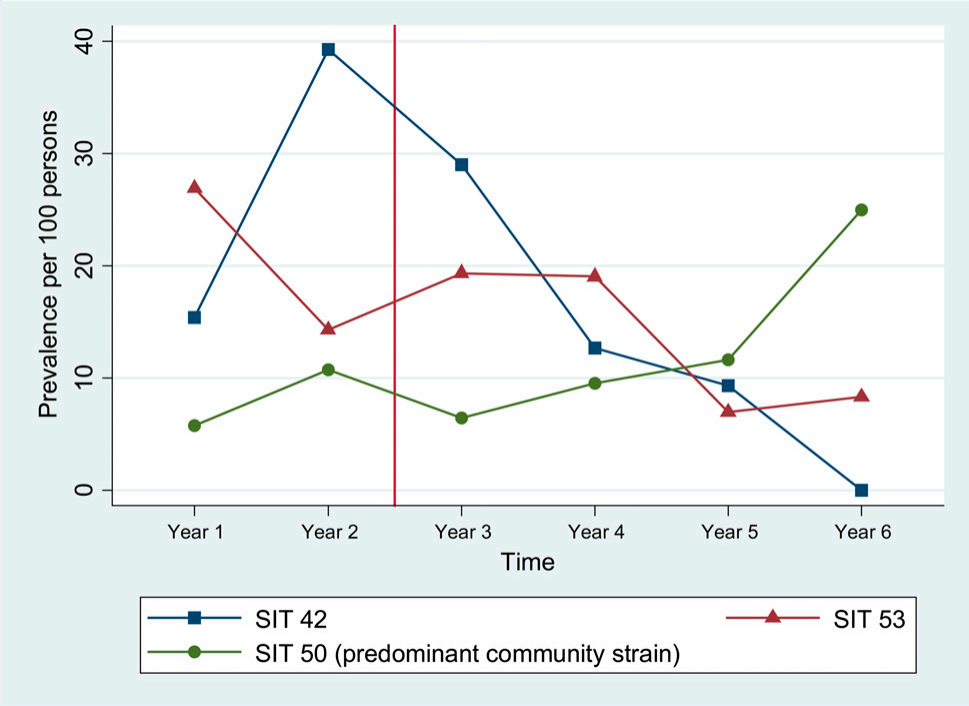
Change in prevalence over time of highly drug-resistant nosocomial outbreak strains SIT 42 and SIT 53 vs. non-drug-resistant predominant community strain SIT 50 in Hospital Dos de Mayo inpatients living with human immunodeficiency virus infection. Intervention marked with vertical line. SIT 42 and SIT 53 highly drug-resistant nosocomial outbreak strains. SIT 50 non-drug-resistant predominant community strain. SIT = spoligotype international type.

**Figure 2.**
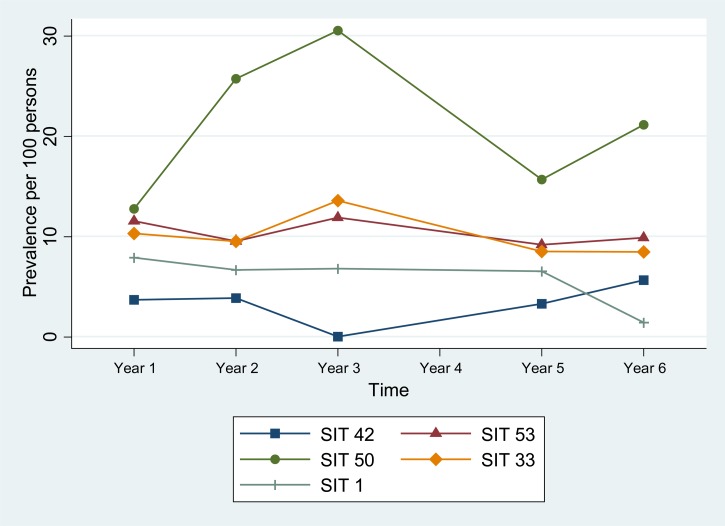
Change in prevalence over time of five common strains in community outpatients without human immunodeficiency virus infection. SIT = spoligotype international type.

In the first year of use of the HDM new isolation ward, prevalence of SIT 42 among inpatients decreased to 29% of cases, and again the next year to 13% of cases. Prevalence continued to decrease in the years that followed, and within 3 years of the intervention, SIT 42 was no longer detected in the HDM cohort (Figure 1

). During this time, prevalence of SIT 42 in the community remained fairly constant (Figure 2

).

## Discussion

The data characterize an outbreak of TB genotype SIT 42 (LAM9 clade), and possibly also SIT 53 (T1 clade), among inpatients in a hospital HIV ward during a 6-year period. The high prevalence of SITs 42 and 53 was not observed with the other TB genotypes circulating in the HDM population, nor in the community outpatients without HIV infection during the same period. Transmission of SITs 42 and 53 decreased substantially after implementation of the new isolation ward. This suggests that these two strains were related to the nosocomial spread of MDR and PZA-resistant TB within the HDM population, and demonstrates the effectiveness of control measures such as negative pressure ventilation, use of rapid diagnostics, and source isolation in this resource-constrained setting.

The two most common *pncA* gene mutations in this study, G145A (D49N PZAse mutation) and A403C (T135P PZAse mutation), were found exclusively in the SIT 42 and SIT 53 isolates, respectively. All isolates with one of these two mutations were shown to be MDR and PZA resistant; unsurprisingly, SIT 42 and SIT 53 were also shown to be strongly associated with MDR and PZA resistance among HIV-positive HDM inpatients. No significant association with drug resistance was found for these two genotypes among HIV-negative community patients nor in any other common TB genotypes regardless of HIV status. MDR and PZA resistance were highly correlated especially among patients living with HIV infection. However, the association between drug resistance and these two SIT genotypes remained significant even after controlling for the confounding of HIV status.

A study using a guinea pig model to examine the airborne infectiousness of TB in the HIV ward at HDM showed that a very small number of MDRTB patients were responsible for the vast majority of TB transmission.^23^ This could explain the rapid and sustained transmission of the drug-resistant SIT 42 and SIT 53 strains within the HIV-positive HDM population compared with other genotypes.

In several large Peruvian hospitals, inpatients with infectious diseases have historically shared a large open ward without engineering for air extraction.^9^ This greatly increases the likelihood of person-to-person TB transmission.^7^,^24^ HDM, the source of all patients living with HIV infection in this study, operated under these conditions until use of a new respiratory isolation ward for TB patients began in 2001, 2 years into this study. This intervention was associated with a significant decrease in rates of MDRTB, from 56% to 20% among HIV/TB coinfected patients (see the accompanying article in this issue). The effectiveness of the intervention is apparent from the rapid, sustained reduction in transmission of SIT 42, culminating in a complete cessation of transmission of this genotype within 3 years of the new ward opening. Transmission of SIT 53 did not decrease immediately nor stop completely before the end of the study. However, odds of both MDR and PZA-resistant infection decreased significantly after the intervention.

Another notable factor is that SIT 53 had a higher prevalence than SIT 42 in the HIV-negative community population throughout the study (10–3.4% of cases, respectively). In fact, community prevalence of SIT 53 was second only to SIT 50 (Harlem3 clade), a mostly drug-sensitive strain. One possible explanation for the slower decrease in prevalence of SIT 53 compared with SIT 42 after the intervention is that new TB infections in HDM before the intervention were mostly due to intrahospital spread of drug-resistant strains, whereas after the intervention, new TB infections were no longer dominated by intrahospital transmission and began to more closely resemble the distribution seen in the community. This would also explain the increase in prevalence of SIT 50 in the HIV-positive HDM population after the intervention: the most common community strain replaced the dwindling nosocomial strains.

An unexpected trait of our spoligotyping data is the relative unimportance of the SIT 1 genotype of the Beijing family. This strain accounted for less than 6% of patients, and showed no association with drug resistance. This is surprising considering the historical association between Beijing family strains and drug resistance.^25^ A differential evolution of drug resistance might be occurring, without being linked to the mentioned genotyping test markers.

To diagnose TB, sputum smear microscopy was mandatory in all patients with respiratory symptoms who could produce sputum. Smear microscopy has advantages of being inexpensive and highly specific. However, it is also poorly sensitive leading to many false-negative results, and it cannot diagnose drug resistance. The MODS assay was also used in this study to determine multidrug resistance. This assay has been shown to be an inexpensive, sensitive, and specific diagnostic option,^15^,^16^,^20^ and the incorporation of TB drugs into the culture medium allows for the determination of antimicrobial sensitivity to rifampin and isoniazid. Recently, it has also been validated for the detection of extensively drug-resistant TB.^26^ The GeneXpert MTB/RIF (Cepheid, Sunnyvale, CA) machine is another potentially useful diagnostic option that detects drug resistance very rapidly.^27^ Hospital TB control measures should incorporate use of at least one of the available rapid tools for TB diagnosis and detection of drug resistance, and use this information to separate patients and to ensure that all patients receive appropriate, effective treatment. The diagnostic tool that is most appropriate for a particular hospital will depend on factors such as budget, available laboratory equipment and personnel, and the characteristics of the patient population.

Limitations of this study include time gaps in data from the HIV-negative community population, which was most evident between years 3 and 4, and missing data (Wayne test administered to only 601 of 794 isolates, *pncA* mutation data only available for 262).

## Conclusions

Lack of effective infection control measures in an HIV ward favored the ongoing transmission of two highly drug-resistant TB genotypes, SIT 42 and SIT 53. The pervading resistance to PZA found to be associated with these two genotypes can be explained by their strong association with G145A and A403C *pncA* mutations, respectively. The installation of a new respiratory isolation ward at HDM for HIV-positive patients with suspected or confirmed TB greatly decreased nosocomial transmission of these drug-resistant TB strains.

We have shown for the first time the utility of mutation typing when combined with resistance testing and spoligotyping in detecting and characterizing TB outbreaks. Our findings also reinforce the importance of implementing hospital infection control measures, including rapid susceptibility testing and patient isolation, to prevent the spread of drug-resistant TB, especially among patients living with HIV.

## Figures and Tables

**Table 1:** Demographic characteristics, drug resistance characteristics, and *Mycobacterium tuberculosis* genotypes among hospitalized HIV-positive patients and HIV-negative outpatients

	HIV positive *n* (%)	HIV negative *n* (%)†	Total *n* (%)	*P* value§
Total patients	241 (30)	553 (70)	794 (100)	
Demographic characteristics				
Age in years, mean (standard deviation)	32 (7.8)	31 (13)	563*	0.4
Age range	18–63	15–78		
Gender			792*	
Female	49 (21)	241 (44)	290 (37)	
Male	190 (80)	312 (56)	502 (63)	< 0.001
Drug resistance characteristics¶				
PZA resistant	64 (34)	30 (7.3)	94 (16)	< 0.001
MDR	82 (35)	53 (9.7)	135 (17)	< 0.001
MDR with PZA resistance	62 (78)	15 (30)	77 (59)	
*pncA* mutation present∥	69 (50)	35 (29)	104 (40)	< 0.001
No. of unique spoligotypes	59 (24)	127 (23)	149 (19)	
SIT (clade) in order of total prevalence‡				
Id. 177 SIT 50 (Harlem3)	25 (10)	105 (19)	130 (16)	0.003
Id. 188 SIT 53 (T1)	41 (17)	57 (10)	98 (12)	0.008
Id. 75 SIT 33 (LAM3)	12 (5)	54 (10)	66 (8)	0.03
Id. 144 SIT 42 (LAM9)	40 (17)	19 (3)	59 (7)	< 0.001
Id. 3 SIT 1 (Beijing)	9 (4)	35 (6)	44 (6)	0.1
Id. 161 SIT 47 (Harlem1)	5 (2)	26 (5)	31 (4)	0.08
Id. 130 SIT 1355 (T2)	11 (5)	13 (2)	24 (3)	0.09
Id. 118 SIT 222 (T1)	6 (2)	18 (3)	24 (3)	0.6
Id. 47 SIT 91 (X3)	7 (3)	15 (3)	22 (3)	0.9
All other genotypes	85 (35)	211 (38)	296 (37)	

HIV = human immunodeficiency virus; LAM = Latino American-Mediterranean TB clade; MDR = multidrug resistant; PZA = pyrazinamide; SIT = spoligotype international type; TB = tuberculosis.

* Missing values (age, *N* = 231; and gender, *N* = 2).

† HIV negative or unknown.

‡ Phylogenetic clade assigned according to spolDB4 Pasteur database, SIT number given when the clade is represented by a unique prototype as defined in SpolDB4 Pasteur database.

§ *P* value for the Pearson χ^2^ proportion test at significance level of (α) 5%.

¶ MDR test performed in 782 isolates, Wayne test for PZA resistance performed in 601 isolates, 187 of which were HIV seropositive and 130 of which were MDR.

∥ *pncA* mutation data available for 262 isolates, 139 of which were HIV seropositive.

**Table 2:** Association between common *Mycobacterium tuberculosis* genotypes and drug-resistant phenotype adjusted for relevant variables

Independent variable	MDR								
	Simple logistic regression*			Multiple logistic regression†			MLR with interaction‡		
	OR	CI	*P* value	OR	CI	*P* value	OR	CI	*P* value
SIT 1 (Beijing)	0.69	0.24–2.0	0.5						
SIT 33 (LAM3)	0.11	0.01–0.80	0.03						
SIT 42 (LAM9)	14	7.30–25	< 0.001	12	6.3–22	< 0.001	2.6	0.83–8.2	0.1
SIT 50 (Harlem3)	0.59	0.29–1.2	0.144						
SIT 53 (T1)	3.4	2.0–5.7	< 0.001	3.8	2.2–6.3	< 0.001			
HIV status	5.0	3.4–7.4	< 0.001	3.6	2.3–5.5	< 0.001	3.1	2.0–4.8	< 0.001
Age	0.99	0.97–1.0	0.2						
Sex	1.5	1.0–2.3	0.045						
HIV/SIT 42 interaction							8.5	1.9–38	0.006
Pseudo *R*^2^				0.20			0.18		
	PZA resistance								
	Simple logistic regression*			MLR†					
	OR	CI	*P* value	OR	CI	*P* value			
SIT 1 (Beijing)	0.36	0.05–2.8	0.331						
SIT 33 (LAM3)	NA								
SIT 42 (LAM9)	15	7.9–30	< 0.001	12	5.9–23	< 0.001			
SIT 50 (Harlem3)	0.89	0.38–2.1	0.8						
SIT 53 (T1)	4.9	2.7–9.1	< 0.001	5.0	2.7–9.3	< 0.001			
HIV status	6.7	4.1–11	< 0.001	4.4	2.6–7.4	< 0.001			
Age	0.99	0.97–1.0	0.3						
Sex	0.94	0.60–1.5	0.8						

CI = confidence interval; HIV = human immunodeficiency virus; LAM = Latino American-Mediterranean TB clade; MDR = multidrug resistant; MLR = multiple logistic regression; OR = odds ratio; PZA = pyrazinamide; NA = not applicable due to model omission, predicts failure perfectly; SIT = spoligotype international type.

* Simple logistic regression using MDR or PZA resistance as dependent variable. An indicator variable for SIT genotype compares each of these five most common SITs against all SITs other than these five.

† Multiple logistic regression using MDR or PZA resistance as dependent variable and SIT 42 and SIT 53 as independent variables of interest, adjusted for HIV status. Model chosen by backwards selection until all variables were statistically significant at level of (α) 5%.

‡ Multiple logistic regression using MDR as dependent variable and SIT 42, HIV, and an interaction term for HIV multiplied by SIT 42.

**Table 3:** HIV status, drug resistance characteristics, and genotype associations of common *pncA* mutations

Protein mutation	DNA mutation	HIV+	%§	†HIV−	%§	*P* value‡	Total	%§	MDR	%¶	*P* value*
D49N	G145A	31	22	1	0.8	< 0.001	32	12	32	100	< 0.001
T135P	A403C	11	7.9	4	3.3	0.1	15	5.3	15	100	< 0.001
Y103Stop	C309G	9	6.5	0	0.0	0.004	9	3.4	8	89	0.02
K48T	A143C	3	2.2	5	4.1	0.4	8	3.1	7	88	0.03
H51R	A152G	3	2.2	4	3.3	0.6	7	2.7	3	43	0.7
All others (25 types, ≤ 3 isolates of each)		12	8.6	21	17	0.04	33	13	25	76	0.002
wild type		70	50	88	72	< 0.001	158	60	41	26	< 0.001
Total (with mutation data)		139	100	123	100		262	100	131	50	
Total mutations		69	50	35	29	< 0.001	104	40	90	87	< 0.001
Protein mutation	DNA mutation	PZA resistant*	%	*P* value‡		SIT 42	%¶	SIT 53	%¶		
D49N	G145A	32/32	100	< 0.001		32	100	0	0		
T135P	A403C	15/15	100	< 0.001		0	0	15	100		
Y103stop	C309G	8/9	89	< 0.001		0	0	6	67		
K48T	A143C	0/7	0	0.054		0	0	0	0		
H51R	A152G	5/7	71	0.035		1	14	0	0		
All others (25 types, ≤ 3 isolates of each)		17/31	55	0.009		4	12	4	12		
Wild type		11/154	7.1	< 0.001		6	3.8	22	14		
Total (with mutation data)		87/255	34			43	16	47	18		
Total mutations		76/101	75	< 0.001		37	36	25	24		

HIV = human immunodeficiency virus; LAM = Latino American-Mediterranean TB clade; MDR = multidrug resistant; PZA = pyrazinamide; SIT = spoligotype international type.

* Missing values (Wayne test data for PZA resistance on 255 of 262 isolates with mutation data).

† HIV negative or unknown status.

‡ *P* value for the Pearson χ^2^ proportion test at significance level of (α) 5%.

§ Percentage of total (with mutation data) row.

¶ Percentage of total column.

∥ Percentage of total column with available PZA resistance data.

**Table 4:** Characteristics of TB isolates and odds of infection before and after implementation of the new TB infection control intervention in Hospital Dos de Mayo

	HDM patients living with HIV infection and receiving intervention							Before new ward		Community patients receiving no intervention§			
	Before new ward			After new ward		OR*	*P* value†			After new ward		OR*	*P* value**
	*N*	%		*N*	%			*n*	%	*n*	%
Drug resistance			Intervention‡										
MDR	40/78	51		42/157	27	0.35	< 0.001	13/270	4.8	40/277	14	3.7	0.04
PZA	35/47	74		29/140	21	0.076	< 0.001	12/173	6.9	18/241	7.5	1.3	0.7
Genotype													
SIT 42 (LAM9)	19	24		21	13	0.51	0.06	10	3.7	9	3.2	0.62	0.5
SIT 50 (Harlem3)	6	7.5		19	12	1.7	0.3	48	18	57	20	0.81	0.5
SIT 53 (T1)	18	23		23	14	0.51	0.07	29	11	28	9.9	1.07	0.9
SIT 33 (LAM3)	4	5.0		8	5.0	1.3	0.7	27	10	27	9.5	0.59	0.2
SIT 1 (Beijing)	3	3.8		6	3.7	0.92	0.9	20	7.4	15	5.3	0.79	0.7
All others	30	38		84	52	1.8	0.04	136	50	147	52	1.6	0.1
Total	80	100		161	100			270	100	283	100		
SIT 42 or SIT 53	37	46		44	27	0.42	0.003	39	14	37	13	0.90	0.8

CI = confidence interval; HDM = Hospital Dos de Mayo; HIV = human immunodeficiency virus; MDR = multidrug resistant; OR = odds ratio; PZA = pyrazinamide; TB = tuberculosis; SIT = spoligotype international type.

* OR comparing odds after to before intervention, adjusted by age and gender, determined by logistic regression.

† *P* value for the OR coefficient for the intervention.

‡ Intervention: use of new TB ward in HDM separating TB and HIV patients began 2 years into the study.

§ HIV negative or unknown status.
